# Repression of Alzheimer's beta-Secretase

**DOI:** 10.18632/aging.100612

**Published:** 2013-10-26

**Authors:** Francesca-Fang Liao, Ruishan Wang, Edwards A. Park

**Affiliations:** ^1^ Department of Pharmacology, University of Tennessee Health Science Center, College of Medicine, Memphis TN 38163, USA; ^2^ Department of Pathology, University of Tennessee Health Science Center, College of Medicine, Memphis TN 38163, USA

Alzheimer's disease (AD) is one of the most devastating neuro-degenerative disorders characterized by the two pathological hallmarks of amyloid plaques and neurofibrillary tangles. Multiple environmental factors, such as diet and life style, along with genetic factors are all significant contributors. In addition, classic cardio-metabolic factors such as hypertension, stroke, diabetes and hypercholesterolemia increase the risk of AD. Epidemiological, clinical and experimental evidence strongly link metabolic defects with functional alterations associated with AD pathogenesis. Amyloid peptides (Aβ), the major constituent of plaques, are generated by sequential proteolytic cleavage of the amyloid precursor protein (APP) via β-secretase (BACE1) and the γ-secretase. The selectively affected regions in the frontal brains of AD patients are often found to not only display high accumulation of Aβ peptides but also aberrant metabolic states (impaired homeostasis of lipid, glucose and energy metabolism) and chronic neuroinflammation. Together, these events trigger ultimate neuronal loss and cognitive deficit. BACE1 is the rate-limiting enzyme in APP processing and Aβ generation and thus remains one of the most favored therapeutic targets for treating AD. BACE1 expression is tightly regulated at multiple levels including transcription, post-transcription, translation and post-translation (Vassar et al. J Neurosci. 2009; 29:12787). As was recently reviewed (Chami et al., Mol. Neurodegener. 2012; 7:52), BACE1 is a stress-inducible protease. Multiple conditions including oxidative stress, inflammation, hypoxia/ischemia and traumatic conditions induce BACE1 expression primarily via transcriptional activation. We have recently reported differential regulation of BACE1 by oxidative and nitrosative signals (Kwak et al. Mol Neurodegener. 2011; 6:17) and by metabolic stress conditions (Wang et al. Cell Metab. 2013; 17:685).

The peroxisome proliferator-activated receptor-γ (PPAR γ) is a prototypic ligand-activated nuclear receptor (NR) that utilizes PPARγ coactivator (PGC-1α) as its coactivator to coordinate lipid, glucose and mitochondrial metabolism. Based on the ability of PPARγ agonists to elicit anti-amyloidogenic, anti-inflammatory and insulin-sensitizing effects, rosiglitazone and several non-steroidal anti-inflammatory drugs (NSAIDs) are being evaluated in AD clinical trials (Heneka et al. Biochim Biophys Acta. 2007; 1771:1031; Landreth et al. Neurotherapeutics._2008; 5:481). We recently investigated this PPARγ-PGC-1 axis in BACE1 regulation under various metabolic stress conditions and made several previously unappreciated observations. We discovered that 1) feeding mice high-fat-diets upregulate BACE1 transcription while fasting suppress BACE1, 2) up- or down-regulation of PGC-1α reciprocally regulate BACE1 transcription, 3) full activity of PPARγ and PGC-1α are required for suppressing BACE1 and both rely on a SIRT1-mediated deacetylation, and 4) the role of PPARγ-PGC-1α in transcriptional repression of BACE1 appears to represent a unique noncanonical mechanism which is not dependent on its ligand rosiglitazone but rather involves recruitment of a corepressor NCoR. To our knowledge, the PPARγ-PGC-1α inhibition of BACE1 gene expression is the first report of direct transcriptional repression by PGC-1α.

**Figure 1:**
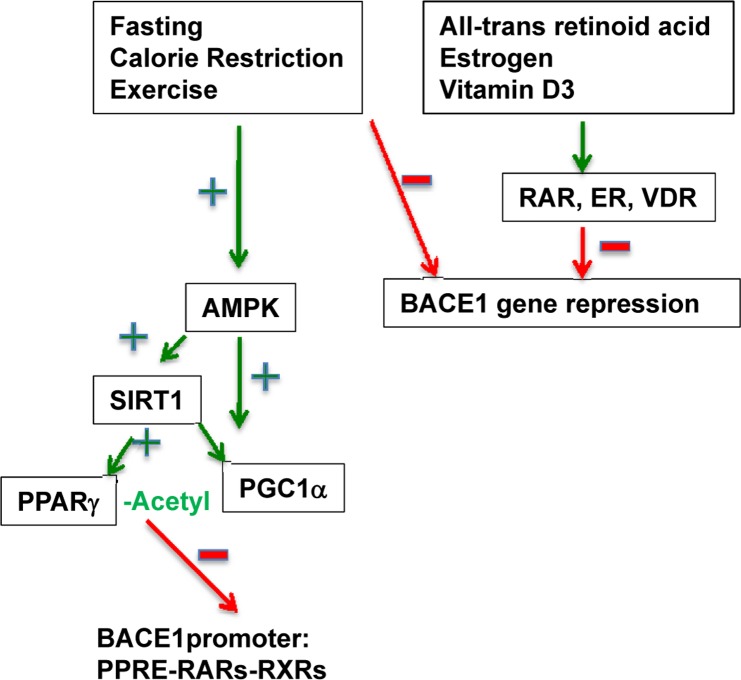
Complex transcriptional repression of BACE1 gene expression by various NR signaling pathways. As shown on left side, calorie restriction leads to activation of AMPK, SIRT1 and PGC-1 and subsequent repression of BACE1 gene expression. At the bottom of the figure, the binding complexes for PPARγ, PGC-1, SIRT1 and NCoR on the PPREs of the BACE1 promoter are shown (Wang, Cell Metab. 2013; 17:685). The binding of other basal transcription factors Sp1, YY1 and HNF-3 are modeled. We speculate that atRA suppresses BACE1 via direct binding of RAR-RXR onto RAR elements that overlap the PPREs. We also predict that estrogen and vitamin D3 mediate their suppressive effects via their respective nuclear receptors ER and VDR through as yet unidentified promoter binding sites. The RAR, ER and VDR may recruit corepressors such as LCoR in a ligand-dependent manner.

Nuclear receptor signaling is increasingly recognized as having important roles in CNS functions (Malaspina, J Neurochem. 2008; 104:584). Classically, NRs are ligand-activated transcription factors which bind to specific responsive elements located in the promoter regions of their target genes and recruit transcriptional coactivators. In contrast, nuclear receptor-mediated repression has been reported as occurring through the recruitment of correpressor complexes (e.g., NCoR/SMRT) to unliganded receptor heterodimers such as PPARγ-RXR or RAR-RXR or LXR-RXR (Perissi et al. Nat. Rev. 2010; 11:109). Since the PPARγ-responsive element (PPRE) overlaps with retinoid acid receptor (RAR-RXR) binding motifs in the BACE1 promoter, we also investigated the potential suppressive role of retinoid acids. Indeed, we found that all-trans retinoid acid (atRA), but not the RXR ligand 9-cis RA, suppressed BACE1 transcription both *in vitro and in vivo*. Furthermore, we have obtained preliminary data demonstrating the beneficial effects of estrogen (E2) and vitamin D3 (1, 25-OH form) in suppressing BACE1 transcription (Wang and Liao, unpublished data). The levels of both vitamin D3 and estrogen are often decreased dramatically in menopausal women, likely contributing to the cognitive decline in a significant portion of these individuals. A correlative relationship between vitamin D receptor (VDR) and estrogen receptor (ER) gene polymorphisms and bone mineral density has been reported in some populations. We speculate that the effects with E2 and vitamin D3 on BACE1 suppression are mediated by their respective nuclear receptors (VDR and ER). We further speculate that interaction between these receptors with ER- and VDR-responsive element(s) may recruit different corepressors such as ligand dependent corepressor (LCoR) in a ligand-dependent manner. Alternatively, the ER and VDR may couple with the NCoR recruited by PPARγ-PGC-1 (Figure 1). A fuller understanding of these NR-mediated regulatory mechanisms in suppressing BACE1 expression is fundamental to the future design of novel therapeutics. Continued study of the detailed interplay between the different nuclear receptor signaling pathways may provide a rationale for selective combination therapy between PPARγ agonists, atRA, vitamin D3 and estrogen to achieve maximum synergistic or additive effects.

